# Grazing deterrents improve survival of outplanted juvenile corals

**DOI:** 10.1007/s00338-025-02703-z

**Published:** 2025-07-05

**Authors:** Eveline van der Steeg, Adriana Humanes, John C. Bythell, Jamie R. Craggs, Alasdair J. Edwards, Yimnang Golbuu, Liam Lachs, Margaret W. Miller, Janna L. Randle, James R. Guest

**Affiliations:** 1 School of Natural and Environmental Sciences, Newcastle University, Newcastle Upon Tyne, UK; 2 KAUST Coral Restoration Initiative (KCRI), King Abdullah University of Science and Technology, Thuwal, Saudi Arabia; 3 Horniman Museum and Gardens, London, UK; 4 Palau International Coral Reef Center, Koror, Palau; 5 The Nature Conservancy, Micronesia and Polynesia, Koror, Palau; 6 SECORE International, Miami, USA

**Keywords:** Fish grazing, Restoration, Coral outplanting, Sexual propagation, Juvenile mortality, Coral seeding

## Abstract

High mortality of juvenile corals hinder both the natural recovery of populations and the successful implementation of restoration efforts. Grazing is a significant cause of juvenile coral mortality, and grazer exclusion devices have been shown to increase juvenile coral survivorship. However, most experiments have used cages that typically alter water flow and light conditions, making it difficult to separate abiotic effects from those of grazing. Here, we test whether grazing deterrents can increase the survival and growth of six-month-old *Acropora digitifera* juveniles outplanted to a shallow reef crest, using arrangements of two or four long or short masonry nails designed to physically exclude larger grazers (e.g., parrotfish) while minimising abiotic changes. By the end of our study, colonies with deterrents had significantly larger planar area (almost tenfold for the most effective treatment), more branches, greater height, and higher survival than those without deterrents. A critical period in this study was the first week after outplanting when colonies with deterrents had significantly less tissue area loss from grazing than those without. Lower area loss in the first week was associated with significantly higher survival over the following 14 months, with an almost threefold improvement for the most effective treatment. For heavily grazed systems, our study highlights the importance of incorporating grazing deterrents into outplant devices to counteract the negative impact of large grazers on newly outplanted juvenile corals and boost restoration success.

**Supplementary Information:**

The online version contains supplementary material available at 10.1007/s00338-025-02703-z.

## Introduction

The recovery and resilience of coral reefs depend on successful coral recruitment after disturbances (Holbrook et al. 2018; Gouezo et al. 2019a). High mortality of coral in early life history stages is a critical bottleneck in this process (Babcock 1985; Wilson and Harrison 2005; Miller 2014; Suzuki et al. 2018). The global decline in coral cover (Hughes et al. 2017; Souter et al. 2020) has led to calls for large-scale assisted recovery via restoration and rehabilitation (Anthony et al. 2017). However, coral outplant success evidenced by outplanted corals reaching reproductive ages has been limited (Boström-Einarsson et al. 2020; Edwards et al. 2024). Asexual fragmentation is currently the most commonly used method to restore degraded reefs (Young et al. 2012; Ferse et al. 2021). However, the low levels of genotypic diversity among fragments, post-outplantation mortality, and challenges in upscaling have been barriers to successful restoration (Omori 2011). The use of sexual reproduction as a method to propagate corals can overcome some of these problems. Substantially higher numbers of outplants with greater genotypic diversity can be produced using such methods, leading to fewer impacts on donor reefs and generating greater upscaling opportunities (Randall et al. 2020; Banaszak et al. 2023). Nonetheless, a major unresolved challenge to implementation is the high level of coral mortality during post-settlement and juvenile stages (Babcock and Mundy 1996; Vermeij and Sandin 2008; Penin et al. 2010; Tebben et al. 2014; Gallagher and Doropoulos 2017). Managing these limitations effectively will determine success or failure within restoration interventions.

Predation and incidental removal by grazing fish can have a considerable negative impact on juvenile coral survivorship (Bak and Engel 1979; Brock 1979; Penin et al. 2010; Lenihan et al. 2011; Doropoulos et al. 2016; Gallagher and Doropoulos 2017). Whether this is due to incidental grazing or targeted feeding to meet dietary requirements (e.g. Clements et al. 2017) is unclear and perhaps varies, but the damage caused and effects on survivorship are well documented (Penin et al. 2010; Doropoulos et al. 2012; Trapon et al. 2013a). Among the major corallivores are parrotfish, butterflyfish, damselfish, *Acanthaster* starfish, *Drupella* snails and occasionally urchins, although a wide variety of fishes and invertebrates are known to feed on coral (Cole et al. 2008; Rotjan and Lewis 2008; Bruckner and Bruckner 2015). While grazing by small fish can be detrimental to juvenile corals (Christiansen et al. 2009; Gallagher and Doropoulos 2017; Neil et al. 2025), it can reduce competition with algae and other benthic invertebrates and remove corallivorous invertebrates, resulting in an increase in juvenile coral settlement and survivorship (McClanahan 1994; Arnold et al. 2010; Webster et al. 2015; Doropoulos et al. 2016; Whitman et al. 2025). Larger fish, however, pose a greater risk to juvenile corals as bioerosion rates increase with fish size, a phenomenon extensively studied in parrotfish (Bruggemann et al. 1996; Bonaldo and Bellwood 2008; Lokrantz et al. 2008). For example, small butterflyfish bites or single parrotfish bite scars on corals often heal over time, whereas larger scars, or focussed biting (extensive removal of tissue and skeleton through repeated, overlapping bites), often do not, leaving damaged areas vulnerable to algal overgrowth (Oren et al. 1997; Bruckner et al. 2000; Welsh et al. 2015; Rempel et al. 2020). The scraping and excavating nature of parrotfish bites is particularly damaging to corals, as it removes not only coral tissue but also the underlying skeleton (Bellwood and Choat 1990; Bonaldo and Bellwood 2009). Larger parrotfish, defined as individuals exceeding 15 to 20 cm in length depending on the species, excavate disproportionately more substrate when grazing than smaller fish, amplifying their impact on juvenile corals (Bruggemann et al. 1996; Fox and Bellwood 2007; Bonaldo and Bellwood 2008; Lokrantz et al. 2008; Ong and Holland 2010). For instance, the excavating bumphead parrotfish (*Bolbometopon muricatum*) and steephead parrotfish (*Chlorurus strongylocephalus*) larger than 45 cm in length*,* exhibit disproportionately high bioerosion rates, further highlighting the substantial impact of large grazers (Bellwood et al. 2003; Yarlett et al. 2018). Predation from parrotfish has caused high mortality of coral outplants, reducing the efficiency of restoration activities (Page et al. 2018; Koval et al. 2020; Rivas et al. 2021; Smith et al. 2021; Knoester et al. 2023). Despite the negative effects of parrotfish on coral outplants, grazers play a crucial ecological role that strengthens coral reef resilience through their suppression of competitive algae (Hawkins and Roberts 2004; Mumby et al. 2013; Shantz et al. 2020). Juvenile coral density and parrotfish biomass are often positively correlated (Hughes et al. 2007; Hoey et al. 2011; Mumby et al. 2013), highlighting the importance of grazers for a healthy ecosystem.

To better understand the factors that determine juvenile coral survivorship, previous studies have focused on physically limiting the access of grazers to corals. However, quantifying the effects of large grazers alone on juvenile coral survival is challenging as most studies use cages or specially designed substrates that exclude nearly all grazers, large and small, which may result in increased algal growth that can compromise coral survivorship. Furthermore, exclusion cages can alter abiotic factors, such as water flow, light, and sedimentation, confounding the effect of grazer exclusion. As a result, grazer exclusion studies have found conflicting results, with some showing that grazer exclusion leads to an increase in juvenile coral survival (Baria et al. 2010; Nakamura et al. 2011; Penin et al. 2011; Trapon et al. 2013b; Whitman et al. 2024) whereas others report a decrease, likely due to intensification of algal growth and competition (Arnold et al. 2010; Steneck et al. 2014; Webster et al. 2015; Doropoulos et al. 2016; Leong et al. 2018). These contradictory results suggest that the effect of grazers on juvenile coral survivorship is highly context-specific and dependent on micro-environmental conditions (Page et al. 2024). For corals outplanted for restoration, adding physical structures designed to deter larger grazers, instead of excluding all grazers, effectively reduced grazing in the first weeks to months after outplanting (Rivas et al. 2021; Pisano 2023; Rule 2023; Dotson et al. 2024; Whitman et al. 2024, 2025), but effects much beyond one year have not been studied.

Faced with increasingly severe and frequent disturbances and continued declines in coral cover, sexual coral propagation appears to be a promising method for restoring degraded reefs at larger scales (Randall et al. 2020; Banaszak et al. 2023). This method can be combined with selective breeding for stress resistance to enhance coral adaptive potential to future climate conditions (van Oppen et al. 2015, 2017; Humanes et al. 2024). However, outplanted sexually propagated corals face similar survival bottlenecks to natural recruits, with high early mortality being a common occurrence (Omori et al. 2008; Guest et al. 2014; Miller 2014; Chamberland et al. 2015, 2017; Humanes et al. 2021; Randall et al. 2021, 2022; Page et al. 2024). One way to increase outplant survival is to rear corals in *ex-situ* nurseries for longer periods to reach an “escape size” prior to outplanting (Guest et al. 2014). However, nursery rearing may not be feasible everywhere as it requires land-based aquarium facilities or suitable locations for ocean nurseries sheltered from storms. If corals can be transplanted to target areas earlier while overcoming juvenile mortality bottlenecks, such as predation, restoration success could increase considerably. Therefore, there is a growing need to design minimally invasive methods to selectively exclude larger grazers that have the potential to harm outplanted corals and compromise their growth and survivorship.

The detrimental effect of large grazers on juvenile corals has the potential to hinder the efficacy of restoration efforts. Despite this, the potential effects of grazing on coral survival beyond the first few months after settlement is relatively unexplored. This study aims to estimate the impact of deterring grazers on the mortality and growth of outplanted corals. We outplanted six-month-old sexually propagated juvenile *Acropora digitifera* colonies to the reef with different grazer deterrent treatments and followed them for 14 months after outplanting (i.e. 20 months age). We studied how the impact of initial grazing deterrence in the first week after outplanting affected survivorship after 14 months on the reef, and aimed to identify potential grazers via video assays.

## Methods

### Study site

This study was conducted in the Republic of Palau in the western Pacific Ocean. Coral spawning and larval rearing were carried out at the Palau International Coral Reef Center (PICRC) in April 2020. A protected outer reef (Mascherchur, N 07°17′29.9′′; E 134°31′08.0′′) on the east of Palau was used as a broodstock source and outplant site. The site was selected for its proximity to PICRC and the high abundance of *Acropora digitifera* at 0.5–4 m depth. Corymbose, digitate and tabular *Acropora* dominated the benthic community of this east-sheltered reef area in Palau, with a benthic cover of approximately 50% hard coral, 10% crustose coralline algae (CCA), 16% turf algae and 0% macroalgae (Doropoulos et al. 2014; Roff et al. 2015, 2018). The herbivore biomass (*Siganidae*, *Acanthuridae* and *Scarinae*) on this reef was low, ranging from 65 to 190 kg/ha, compared to 650 kg/ha at some western sites of Palau, with parrotfish being the most abundant group making up approximately 60% (measured from 2012 to 2018 at sites < 1 km away) (Roff et al. 2018; Puk et al. 2020). More recent studies observed that the reef fish biomass on the nearest eastern outer reef (< 1 km) was also low at 90 kg/ha with an herbivore biomass of 30 kg/ha in 2017 (Gouezo et al. 2019b; Muller-Karanassos et al. 2021). However, herbivore biomass has improved between 2017 and 2021, with an increase in mean length for most fish (Muller-Karanassos et al. 2023). All research was conducted under National Marine Research Permits RE-20–04 and RE-21–02.

### Coral rearing

On April 3rd, 2020, five days before the full moon, ten gravid (i.e., containing visible pigmented oocytes) *A. digitifera* colonies with diameters > 20 cm were collected on scuba using a hammer and chisel. Colonies were transported to PICRC and maintained in a 760 L shaded outdoor holding tank. The tank was provided with a continuous flow of 50 µm filtered seawater (FSW) and two magnetic pumps, both attached to three flow accelerators to ensure water movement (Pondmaster 1200 GPH, Accel Aquatic Vortex). The tank was lit with four LED aquarium lights (122 cm Reef Bright XHO 50/50). Every night after collection until spawning, the inflow and pumps were turned off after sunset (19:00) for approximately 150 min (21:30 h), and the tank was covered to block incident light. Setting was observed on April 6th, two days before the full moon, in three colonies and on April 7th in seven colonies at 19:45 and the colonies spawned at 20:30. Coral gamete collection, larval rearing and settlement followed standard coral propagation methods (Guest et al. 2010), further details can be found in the Supplementary Information (Text S1). Gametes were pooled for fertilisation each night and the cross from the first night was used for this study.

Larvae were offered previously conditioned seeding units (SUs) in two 45 L settlement tanks, each containing 500 SUs, at a density of 25 larvae per SU. Ceramic SUs (Ocean Wonders LLC, 19 mm in diameter with a 17 mm long stem) were biologically conditioned with fresh CCA fragments collected from the study site in four 180 L flow-through tanks, each with two pumps (Hydor Koralia Nano 240 Circulation Pump/Powerhead), for six months prior to the experiment. After settlement, SUs were divided among four shallow 180 L *ex-situ* nursery tanks with ~ 4 L/min flow-through 50 μm FSW. Each tank had two aquarium lights (Reef Brite, 48′′ 50/50 white and blue XHO LED; 200 μmol photons m^−2^ s^−1^ over a 12:12 h diurnal cycle) and two pumps (Hydor Koralia Nano 425 Circulation Pump/Powerhead) to ensure water circulation. Fragments from the parental colonies were added to the tanks to provide a source of Symbiodiniaceae for newly settled corals. Eleven days after larvae were introduced to SUs, the upper surfaces of 26 SUs per settlement tank were examined under a dissecting microscope to count the number of settled corals. The mean settlement was 11.3 ± 0.8 corals per SU (4.0 ± 0.3 per cm^2^), resulting in a settlement success of 45 ± 3.0% (mean ± standard error). Numerous grazers were added to each tank to control algal growth (i.e. juvenile rabbitfish, cerithid snails, trochus snails and small filefish), and nursery tanks were cleaned biweekly. Corals were fed thrice weekly with 0.75 g Aqua Core Coral Fusion plankton powder per tank. Most settled SUs (91%) had at least one live coral after approximately five months (162 days) in the *ex-situ* nursery.

### Coral outplant with grazing deterrents

**Fig. 1:**
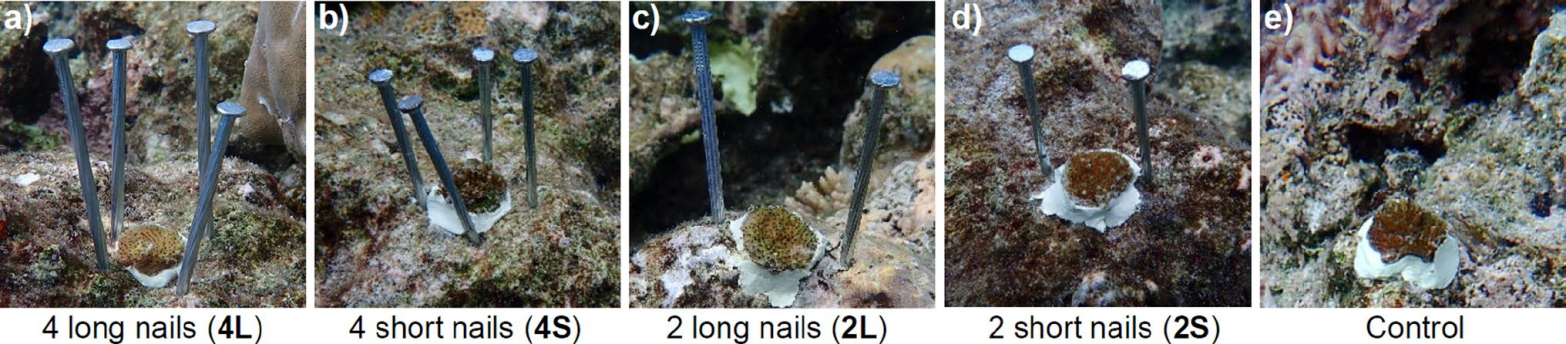
Representative photos of the different levels of grazer deterrent treatments added to juvenile coral colonies outplanted six months after settlement. The colonies had a mean diameter of 2.2 ± 0.25 cm

Experimental treatments were designed to deter larger grazers, while allowing access to smaller algae-grazing fish and minimising alteration of abiotic factors such as water flow, light and sedimentation. Six months (185 days; Oct 2020) after settlement, the SUs with the largest juvenile colonies were selected (n = 200 colonies); these had a mean diameter of 2.2 ± 0.25 cm and planar area of 3.88 ± 0.87 cm^2^. These colonies were outplanted to Mascherchur Reef at 20 cm intervals along four 10 m transects, resulting in 50 corals per transect. To attach each colony, a hole was drilled into the bare reef substrate using a battery-powered submersible drill (Nemo Divers Drill) with an 11 mm drill bit, the substrate brushed clean of algae, and the stems of the SUs secured into the drilled holes with epoxy (Milliput Standard). Using nails arranged around the outplanted corals, five grazing deterrent treatments were applied to the outplants: (a) 4 long (10.1 cm) masonry nails, (b) 4 short (7.6 cm) nails, (c) 2 long nails, (d) 2 short nails and e) a control with no deterrents (n = 40 per treatment, Fig. 1), hereafter referred to as 4L, 4S, 2L, 2S and control respectively. The mean protrusion of long and short nails after attachment to the substrate was 7.1 ± 0.9 cm and 5.1 ± 0.7 cm, respectively. The mean distance between the heads of the nails (measured diagonally for the 4L and 4S treatments) was 5.3 ± 2.1 cm.

SUs were surveyed at 7, 35, 62, 98, 140, 160, 227, 341 and 425 days after outplanting. During surveys, the status of each coral was classified as either alive, missing (SU missing or not found) or dead (entire coral removed from the SU, or intact skeleton without tissue) and any missing nails were replaced. The nails became overgrown with CCA and turf algae during the experiment but remained free from macroalgae. Colony planar area was measured from photos taken at outplanting and at 7-, 160- and 425-day surveys using ImageJ (1.48v, National Institutes of Health, USA). During the last survey, the height of each colony was also measured with callipers and the number of branches counted.

### Fish grazing estimation

To assess the reduction in grazing provided by grazing deterrents, grazing pressure was estimated with video assays. Three replicate sets of five SUs with corals were outplanted in April 2022, 18 months after the start of the original experiment. Corals from the same cohort with sizes comparable to the initial size of outplants (mean diameter of 2.4 ± 0.06 cm) were used. They had been maintained in nursery tanks for 18 months, moved to *in-situ* nurseries for five months where they were grazed and reduced in size, and taken back to the *ex-situ* nursery for one month before the outplant. The *in-situ* nursery was located 2.2 km to the west of the study site on a sheltered sand patch (N 07°18′19.8’’; E 134°30′6.70’’) surrounded by arborescent *Acropora* thickets (see Humanes et al. 2021). SUs were outplanted approximately 20 cm apart, and the treatment order was randomised, with a minimum distance of 20 m between each set. GoPro cameras were deployed at each set of outplants one to three times per day between 9:00 and 16:00 on days three, four and five after outplanting. No divers were in the area after deployment, and the first three minutes of each recording were disregarded to eliminate the effect of camera deployment on fish behaviour. This resulted in 21 videos (49 to 116 min long) and a total of 27 h 40 min of footage. Every fish observed in the recordings that took a bite directly on a coral outplant was identified to species. Its length was estimated to the nearest 5 cm category (5, 10, 15 or 20 cm), using a ruler displayed above the outplants at the start of each video as a guide. Finally, the total number of bites per treatment was recorded. A grazing event was defined as a sequence of consecutive bites on an outplanted coral by a single fish without the fish leaving the frame.

### Data analysis

Differences in survival curves between treatments were compared using Kaplan–Meier survival analysis with right-censored data (Lee and Wang 2003). This method is robust for non-normally distributed survival data and allows the inclusion of individuals we could not relocate as right-censored data. When a coral was found to be entirely dead in a survey, the date the coral died was estimated as the midpoint between surveys, as the exact time of death cannot be determined. Log-rank pairwise comparisons were performed to test for significant differences in survival curves. The effect of treatment (five-levels fixed effect) on planar colony area and colony height were tested separately using Generalized Linear Mixed effects Models (GLMM, Bates et al. 2015) with a log link gamma distribution, accounting for variability between transects (four-level random factor). Residual diagnostics indicated a slight deviation from uniformity for both the area and height models (Kolmogorov–Smirnov test, p = 0.04). However, residual plots revealed no strong patterns, and tests for overdispersion and outliers were non-significant. This minor deviation appeared to result from the inclusion of transect as a random effect, which explained negligible variance and led to a singular fit. A simplified model excluding transect produced comparable fixed-effect estimates; therefore, the full model was retained for consistency and completeness. The number of branches per colony was compared between treatments with descriptive summary methods as the percentage of live colonies that had branches.

For the initial area loss, the percentage of planar colony area reduction during the first week after outplant, dead corals were included as having an area of 0 cm^2^. The effect of treatment (five-level fixed effect) on initial area loss was tested using GLMM (Brooks et al. 2017), with a gaussian error distribution and identity link accounting for differences between transects (four-level random factor). To account for heteroscedasticity, treatment was included as a predictor in the dispersion formula. Diagnostic checks indicated minor within-group residual deviations for transect, but residuals were symmetrically distributed with no clear patterns, indicating the model was appropriate. The effect of initial area loss on survival after 14 months was tested using a GLMM with a binominal error distribution with treatments and transects as random effects. To analyse the effect of treatments on bite rate, the bite rates of all fish species were grouped and adjusted to the number of bites per hour and rounded to the nearest whole number. The effect of each grazing deterrent on fish bite rates was tested using a GLMM with a negative binomial error distribution with plot as random effect (three-level random factor), as data distribution was overdispersed when first run with a Poisson error distribution.

All pairwise comparisons among treatments were conducted using a post hoc Tukey Test with the ‘emmeans’ package (Lenth 2017). Model validation steps included assessing homogeneity of residuals versus fitted values, over- and under-dispersion and a simulation study to test the ability of the model to capture zero-inflation using Q-Q plots and the DHARMa package (Hartig 2018). Results were plotted with the ‘ggplot2’ package (Wickham 2016), and all data analyses were performed in R 4.2.1 using Rstudio 554 (Rstudio Team 2010; R Core Team 2022).

## Results

### Survival and size

**Fig. 2:**
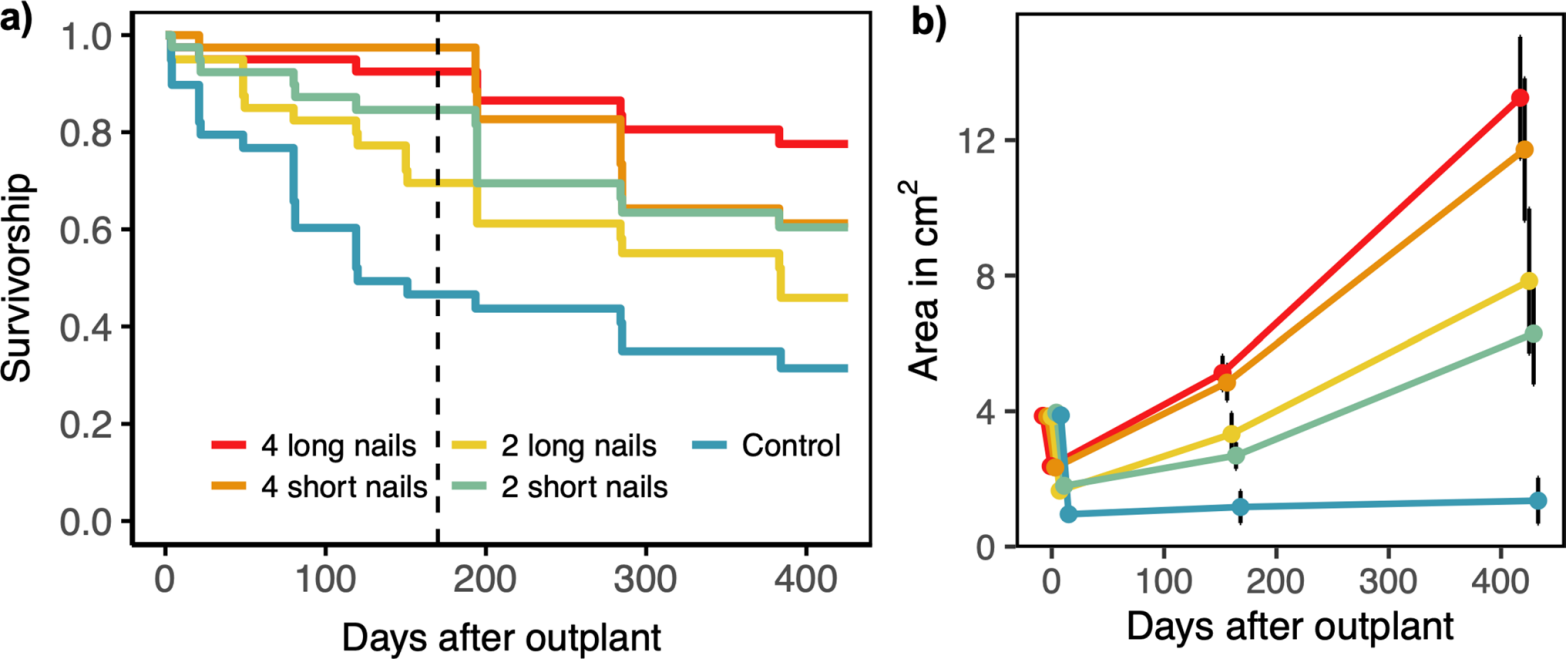
**a** Survival curves of six-month old *Acropora digitifera* outplants with five different levels of grazing deterrents. The dashed line indicates the timing of Typhoon Surigae. **b** Mean area ± SE of colonies in the five treatments at four timepoints in the study. The data points are offset on the x-axis for readability

The mean survival time (days until mortality) of corals within all grazer deterrent treatments, except for 2L, was significantly higher compared to the control after 14 months (425 days) on the reef (*P* < 0.001, *P* = 0.005 and* P* = 0.035 for 4L, 4S and 2S respectively, Fig. 2a, Supplementary Table S1). The 4L treatment also had a significantly longer survival time than the 2L treatment (*P* = 0.042). The mean survival time of corals outplanted to the reef with the 4L grazing deterrents was 374 ± 18 days, 1.8 times longer than the control 212 ± 28 days. Results are given as mean values ± standard error of the mean. The 4L treatment had the highest survivorship after 14 months, with 65% of the colonies alive; this was almost three times higher than the control, with only 22.5% still alive. The actual percentage of live colonies was lower than displayed in the Kaplan–Meier survival curves, as missing outplants (e.g. due to rock falls) were censored, and only included until the last time monitored as alive.

On April 18th, 2021 (192 days after outplant), typhoon Surigae passed north of Palau, causing high wave energy at the study site. Before typhoon Surigae, at approximately one year old (160 days on the reef), 92% of outplants in the 4L treatment were still alive. Of those colonies still alive one month before typhoon Surigae, 22% had died or were missing in the following survey one month after the typhoon. Before the typhoon, the number of nails replaced during surveys was minimal, 0.5% after the first week and averaging 6.7% up to 160 days of the study (Supplementary Table S2). However, after the typhoon a larger proportion of nails were missing, requiring the replacement of 54.4% of them.

**Fig. 3:**
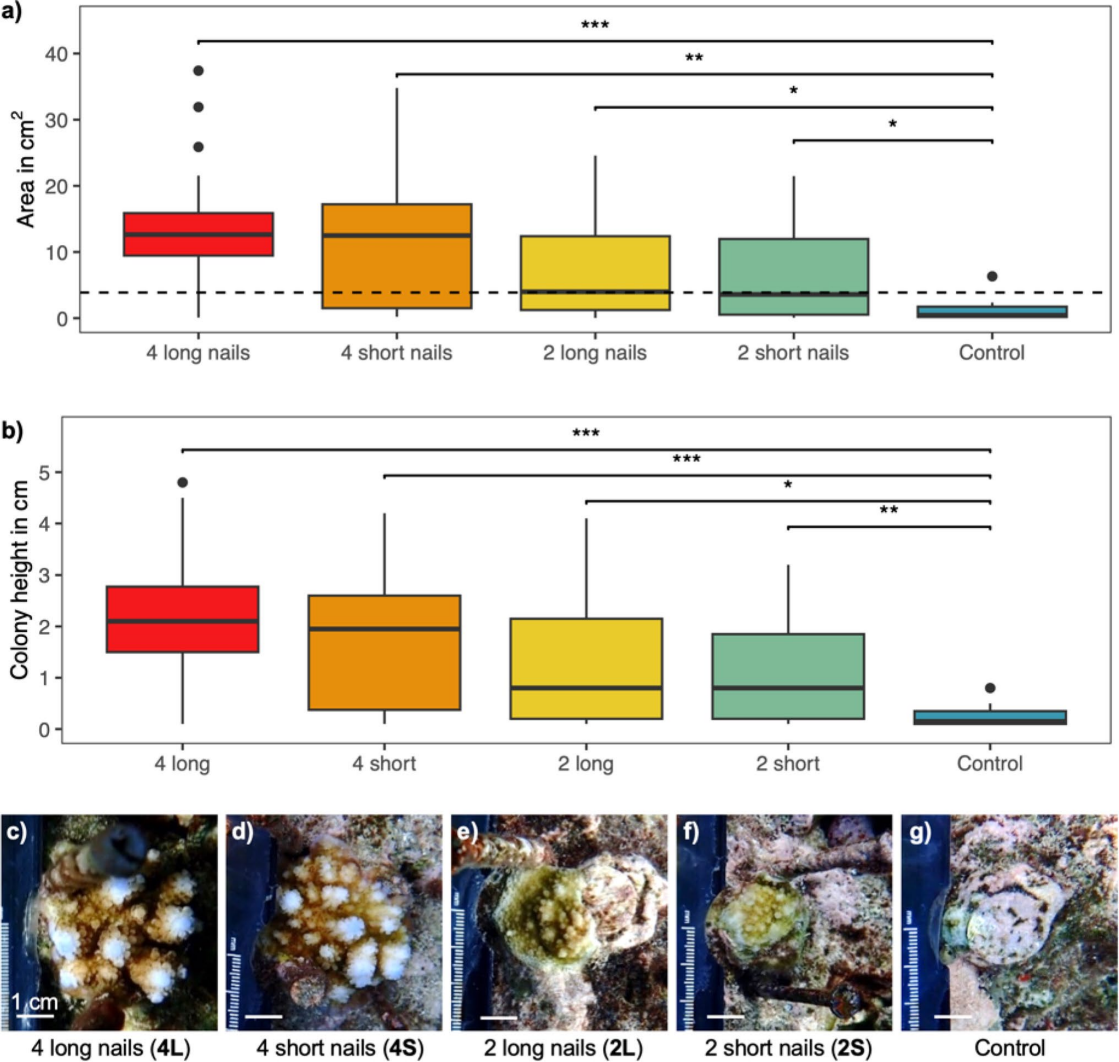
Boxplots of **a** planar area in cm^2^ and **b** colony height of *Acropora digitifera* juvenile corals outplanted at age six months under five different levels of grazing deterrence after 14 months on the reef. The boxplots represent the interquartile range, the horizontal line in the box is the median and the whiskers show the minimum and maximum data values (1.5 * interquartile range) and the variability compared to the interquartile. The black dots are outliers outside of the whisker lines (> 1.5 * interquartile range from the median). The dashed line in 3a is the mean size of all colonies at time of outplant. The effect among treatments was compared with a GLMM with gamma log-link distribution and a post hoc Tukey Test (*** *p* < 0.001, ** < 0.01, * < 0.05). **c**–**g** Photos of representative colonies with areas close to the median size of each treatment, with scale bars of 1 cm at the end of the study

The outplanted colonies experienced a reduction in area within one week after being outplanted to the reef (Fig. 2b). The 4L & 4S recovered fairly quickly, reaching a mean area greater than their initial outplant size within 160 days after outplanting. After 14 months on the reef, the planar area and height of colonies in all grazing deterrent treatments was significantly larger than colonies in the control treatment (GLMM *p* < 0.05, Fig. 3a and b, Supplementary Tables S3 & S4). The mean planar area at the final survey was 13.3 ± 2.5 and 11.7 ± 2.7 cm^2^ for the 4L and 4S treatments, respectively (approximately 3.8 and 3.4 cm in diameter, Figs. 2b, 3c–g). The mean planar area of the 2L and 2S treatments and control was 7.8 ± 2.1, 6.3 ± 1.5 and 1.4 ± 0.4 cm^2^ (approximately 3.2, 2.8 and 1.3 cm in diameter respectively). The percentage of live colonies branching was 77, 70, 47, 35 and 0% for the 4L, 4S, 2L, 2S and control treatments, respectively. The percentage of live colonies self-attached to the substrate (i.e., coral tissue growing directly on the reef substrate) was 63, 55, 40, 43 and 15%, respectively.

### Area loss in the first week post-outplant

**Fig. 4:**
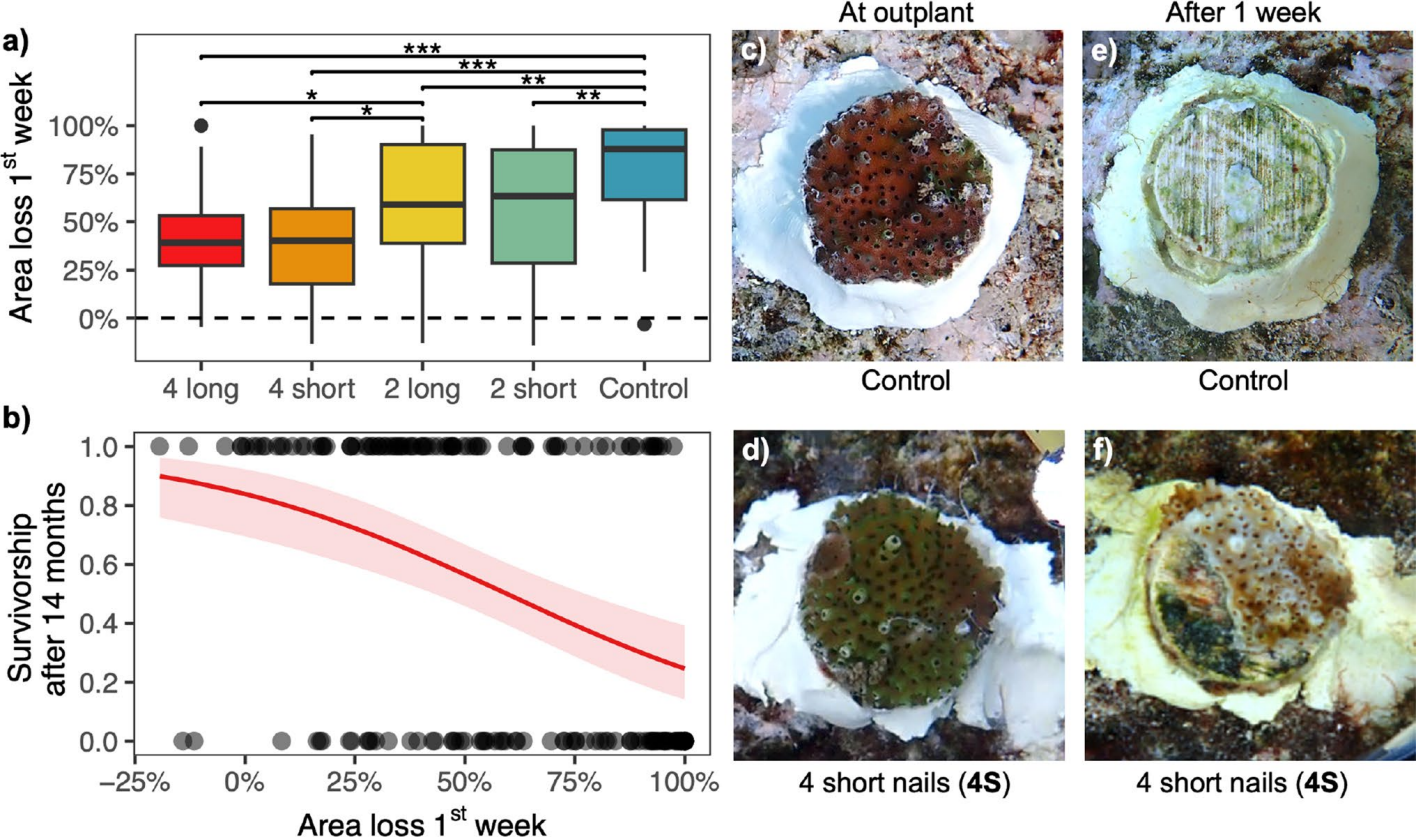
**a** Percentage of planar colony area reduction during the first week after outplant, asterisks indicate the level of significance between grazing deterrent treatments based on a gaussian GLMM and post hoc Tukey test (*** *P* < 0.001, ** < 0.01, * < 0.05). The dashed line indicates 0% area loss. **b** The predicted relationship between percentage loss of planar colony area within one week after outplant and survivorship after 14 months on the reef from a binominal GLMM. The shaded area indicates the confidence interval. **c** Colony from the control treatment with no grazing deterrents at outplant and **e** the same colony after one week on the reef, grazing scars are visible on the seeding unit, and the coral is missing. **d** Colony from the 4 short nail treatment at outplant and **f** the same colony after one week on the reef with part of the colony missing

There was a large effect of grazing on planar colony area loss within the first week after the colonies were outplanted (Fig. 4a, Supplementary Table S5). Colonies without any grazing deterrents (controls) had a mean reduction in size of 78 ± 4%, which was significantly higher than all other treatments (GLMM *P* < 0.01, Fig. 4a, c and e). There was also significantly less area loss in the 4L and 4S treatments compared to the 2L treatment (GLMM *P* < 0.05). Nonetheless, all treatments suffered a considerable reduction in mean area in the first week, with even corals in the 4L and 4S treatments losing up to 40% of tissue area (Fig. 4d and f). Across all treatments, there was a negative effect of initial area loss on long-term survival (Fig. 4b, GLMM, *P* < 0.001, estimate = -2.77, *Z*-value = -4.35, Supplementary Table S6).

### Fish grazing estimation from video

**Fig. 5:**
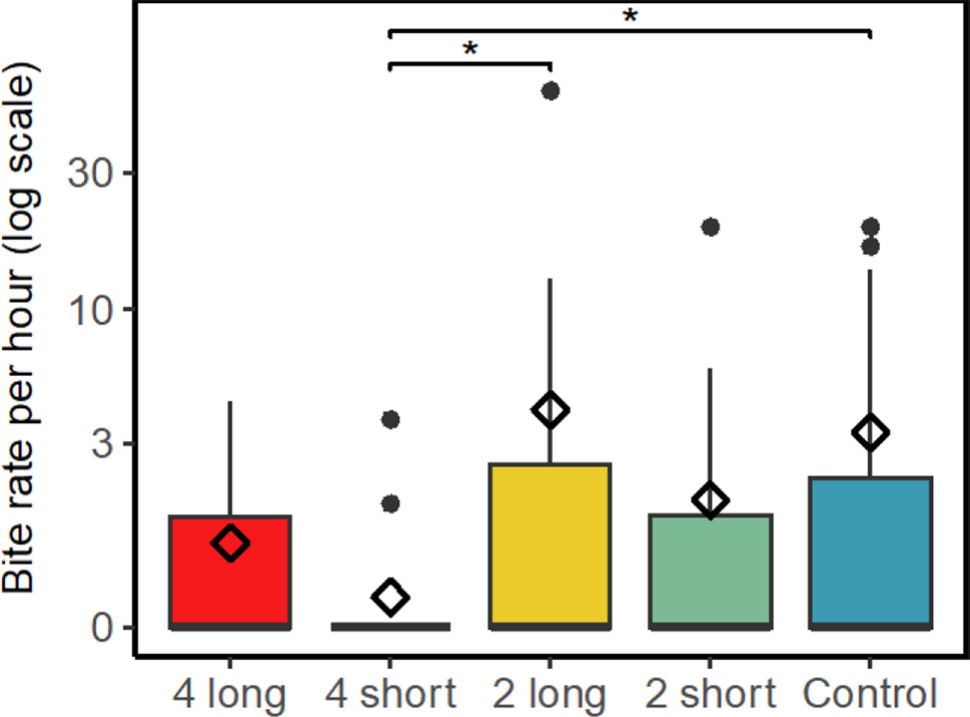
Bite rate recorded as the number of bites per hour of all fishes grouped, tested using a negative binomial GLMM paired with a Tukey Test for pairwise comparisons, plotted on a log scale. Diamonds represent the mean bite rate per hour. Asterisks indicate level of significance between grazing deterrent treatments (*P < 0.05)

A total of 77 grazing events (a sequence of consecutive bites on an outplanted coral by a single fish without the fish leaving the frame) were recorded, with 290 individual bites by 14 species. There was a significant difference in bite rates between the 4S treatment and both the 2L treatment and control (Fig. 5, GLMM with post hoc Tukey Test, *P* < 0.05 for both, Supplementary Table S7). Most bites were taken by *Labrichthys unilineatus,* followed by *Chlorurus spilurus*, *Chaetodon baronessa* and *Ctenochaetus striatus*, 163, 62, 24 and 11 bites from a wrasse, parrotfish, butterflyfish and surgeonfish respectively (Supplementary Figure S1). No schools of grazing fish were observed around the outplants, and no fish over 20 cm were seen grazing on the colonies. Only one fish of ~ 20 cm was observed grazing on a colony in the control treatment and all other fishes were ~ 15 cm or smaller (Supplementary Table S8). No grazing scars were observed on the colonies after the three days of recording.

## Discussion

Survival bottlenecks in the early ontogeny of corals can hinder both the recovery of wild populations following disturbances and the success of coral restoration efforts (Holbrook et al. 2018; Gouezo et al. 2019a; Banaszak et al. 2023). Grazing is known to be a potential moderator of these juvenile survival bottlenecks, as scraping can damage small, settled corals (Penin et al. 2010; Trapon et al. 2013a; Doropoulos et al. 2016). However, the specific influence of large grazers on juvenile coral growth and survival warrants further exploration. Using a manipulative field experiment, we found that the addition of grazing deterrents significantly increased the survivorship and growth of outplanted juvenile corals. Grazing impacts within the first week after outplanting to the reef caused an immediate bottleneck in survivorship and growth for those juvenile corals affected, with lasting effects on the long-term survivorship of even those corals that were still alive after one week. For heavily grazed reefs, our results suggest that simple devices to deter grazing on newly outplanted corals could mitigate juvenile survival bottlenecks and increase the success of restoration efforts.

In our study, juvenile corals suffered from planar area loss and subsequent mortality, particularly in the first week after outplanting (Fig. 4). We were not able to provide direct evidence of which fish species and sizes were excluded, making it impossible to infer which other coral grazers, fish or invertebrates, might have been excluded by the grazing deterrent treatments. It is likely larger fishes were physically excluded, but we cannot unequivocally attribute the effects shown to larger fishes without the data to demonstrate this. The high abundance of parrotfish at the study site, with regular sightings of bumphead parrotfish schools, suggests that a plausible explanation for the high mortality and loss of tissue area in the outplants is grazing by large parrotfish (Roff et al. 2018; Muller-Karanassos et al. 2021; Friedlander et al. 2023; Muller Karanassos et al. 2023). Large parrotfish are known to consume live corals, leaving distinctive grazing scars (Bonaldo and Bellwood 2009; Bonaldo and Rotjan 2018). After observing that most area loss of the outplants occurred during the first week, video assays were set up on a new set of outplanted colonies to identify the types of fish responsible for the damage. The assays showed a significantly lower mean bite rate in the 4S treatment, indicating this treatment was effective at deterring grazers. In addition, small fish were observed to access and graze on coral colonies with grazing deterrents without leaving visible grazing scars. However, the number of grazing observations in the assays was limited, and few large grazers were captured grazing the corals during these relatively short video survey periods. It is possible that the duration of the video assays was not long enough to capture sporadic intensive grazing events by large groups of parrotfish or other grazers that could be a major driver of the juvenile survival bottleneck immediately after outplanting.

A diverse range of grazing, scraping, and corallivorous organisms are associated with coral reefs. Although large parrotfish are the most common coral-scraping fish on tropical reefs, triggerfish and pufferfish have also been observed grazing on coral (Bellwood et al. 2006; Palacios et al. 2014; Gallagher and Doropoulos 2017). In addition, *Acanthaster* starfish and *Drupella* snails can be devastating to coral populations, especially after bleaching events (Cumming 1999; Kayal et al. 2012; Bruckner et al. 2017), and nocturnal invertebrates, such as urchins, are known to graze juvenile corals (Bak and van Eys 1975; Glynn 1996; Korzen et al. 2011; O’Leary et al. 2013). This complicates the survey logistics required to identify the taxa affecting colony growth and survivorship. However, physical grazing deterrents are likely effective for multiple grazer and predator groups. Moreover, reefs with different characteristics, such as high sedimentation or algae cover, likely have additional factors affecting juvenile coral survivorship (Trapon et al. 2013b; Doropoulos et al. 2022). Thus, site-specific trials should be conducted before large-scale coral outplanting efforts are designed and implemented. While abiotic effects of the grazing deterrents on coral mortality and growth were not measured in this study, they were likely negligible. The nails used as grazing deterrents had a minimal profile and were unlikely to decrease water flow or light, nor did they cause any observable algal accumulation on surrounding benthos as they allowed access to intermediate- and micro-herbivores (Carpenter 1986; Altman-Kurosaki et al. 2018).

Fish grazing on newly outplanted corals has the potential to jeopardise the success of coral restoration efforts. Fish grazing on recent transplants has been widely reported (Miller and Hay 2010; Horoszowski-Fridman et al. 2015; Page et al. 2018; Koval et al. 2020; Smith et al. 2021; Whitman et al. 2024). Newly transplanted corals might be more palatable to fish grazers as they likely have increased nutrient content as a result of being fed in the nursery and different Symbiodiniaceae populations compared to wild populations (Leal et al. 2014; Horoszowski-Fridman et al. 2015; Gantt et al. 2023). Stress from transplantation and acclimatisation to the new harsher reef environment (e.g., higher light and water flow conditions) can reduce the performance of corals after outplant (Forrester et al. 2012). Corals in *ex-situ* nurseries tend to be acclimatised to low light conditions (Gantt et al. 2023), often having darker pigmentation than wild colonies. This darker pigmentation is lost several weeks to months after outplanting as the transplants acclimatise to their new environment (Figs. 4e and f, 3d; Horoszowski-Fridman et al. 2015; Page et al. 2018). Another cause of fish grazing on new transplants could be neophilia (i.e., the attraction of grazers to novel objects in the reef) as they explore new food sources. However, continued fish grazing after the first weeks has been observed (Rivas et al. 2021; Knoester et al. 2023; Rule 2023), indicating that fish habituation to novel objects does not reliably preclude predation and incidental damage. Another possibility is that new colonization of turf and other biofilm directly on the epoxy around the outplant could have also been an attractive as a food source for grazers. Further research is needed to investigate the underlying factors driving grazing on newly outplanted corals. However, data from our and other studies, including terrestrial reforestation and vegetation restoration reviews, suggest that long-term protection of outplants from grazing and herbivory is beneficial (Redick and Jacobs 2020; Xu et al. 2023).

Some colonies were grazed to small sizes during the first week and did not recover beyond their initial size at outplant (Fig. 3a). Small colonies generally have lower survivorship than larger ones (Raymundo and Maypa 2004; Vermeij and Sandin 2008; Ligson et al. 2022). This bottleneck can extend until corals reach a size threshold, after which mortality stabilises (Babcock and Mundy 1996; Guest et al. 2014). If grazing is spatially random, a lower probability of grazing on smaller colonies is expected, but given the size of the disturbance from parrotfish bites, it is more likely to encompass the entire colony of smaller rather than larger corals (Hughes and Jackson 1985). Hence, small colonies are more likely to experience whole-colony mortality following direct damage (Bak and Engel 1979). In addition, small remnants of grazed colonies may have insufficient energy reserves for wound healing and defence against competing benthic organisms (Connell 1973; Oren et al. 2001).

For the initial outplanting in our study we used the largest (and hence fastest growing) juvenile colonies. Therefore, there is a chance that these colonies may have been the most fleshy or palatable colonies from the range of colonies available. This could have exacerbated overall grazing pressure and caused an overestimation compared to a study using the slowest growing corals. Furthermore, for the video grazing detection component of our study, the colonies tested had been moved between the nursery and reef multiple times, and thus possibly stressed due to handling. Given the different experimental treatment of these corals compared to those outplanted initially, plus the fact that both studies were conducted at different times for logistical reasons, it is possible that grazer responses were not comparable. This highlights the need for further research to identify the factors contributing to mortality of outplanted juvenile corals.

Successful coral reef recovery or active restoration both require corals to pass the juvenile survivorship bottleneck. Active restoration may be increasingly important in maintaining ecosystem function as coral reef degradation continues (Knowlton et al. 2021; Suggett et al. 2024). Large grazers such as parrotfish are essential for coral reef resilience, but their detrimental effect on juvenile corals and coral outplants can hinder the success of coral reef restoration efforts (Koval et al. 2020; Smith et al. 2021; Lirman et al. 2022). Grazing deterrents can help overcome this bottleneck in areas with significant biomass of grazing fish (Steneck et al. 2014; Rivas et al. 2021; Whitman et al. 2024). On reefs where there are few grazers (e.g., due to overfishing), there may be fewer benefits from grazing deterrents, or they may not be needed at all. Although manually adding deterrents to outplanted corals using the approach in this study would not be realistic for large-scale restoration efforts due to the dive time required for installation, it could provide a cheap and accessible method to test if grazing deterrents are required during the design stages of coral transplantation efforts. Even deterrents with reduced height were effective at improving juvenile coral survival in our study, suggesting that coral settlement substrates with integrated grazing deterrents could be particularly useful. For instance, some recently developed designs showed that grazer exclusion could improve survival by a factor of 1.2–2 times (Page et al. 2024; Whitman et al. 2024), which is similar to the survival gains of 1.5–2.5 found in our study. While relative survival benefits of grazer exclusion were similar, in absolute terms the improvement was higher in our study (15–43% increase in survival over 14 months) compared to Whitman et al. (2024) (15% increase in survival over 8 months). Alternatively, a modular grazing deterrent could be designed to be deployed at outplant, and if grazing pressure is alleviated several weeks or months later, retrieved and reused. Grazing deterrents can, as demonstrated here, increase juvenile survivorship and should be considered as part of coral restoration strategies using sexually propagated corals.

## Supplementary Information

Below is the link to the electronic supplementary material.

Supplementary file1 (DOCX 75 kb)

## Declarations

### Data availability

The data will be made available upon request.
