# Supplementary material for: Grazing deterrents improve survival of outplanted juvenile corals
Source: Coral Reefs. 2025 Jul 5;44(4):1389–401. doi: 10.1007/s00338-025-02703-z (PMC12304057; doi:10.1007/s00338-025-02703-z)
Supplement: Supplementary file 1 — Supplementary file1 (DOCX 75 kb) [file 338_2025_2703_MOESM1_ESM.docx]

**Supplementary Information**

**Text S1**

Gamete bundles were collected from the surface with plastic cups and placed into a 100 L tank with 0.2 µm UV-treated FSW. The water was gently stirred to break the remaining bundles, after which they were left undisturbed for 45 minutes to allow for optimal fertilisation. After stirring, six 20-ml aliquots were taken and counted; these showed that approximately 874,000 eggs had been released. After fertilisation, an additional six 20-ml aliquots were collected and counted; these showed an achieved fertilisation rate of 94.6 ± 0.8 % (mean ± SE). The fertilised embryos were skimmed off the surface and added to a 100 L tank with 0.2 µm UV-treated FSW to wash off the sperm, a process that was repeated twice. Embryos were then added to two 45 L tanks at a density of one larva per 3 ml; the surplus was returned to the ocean. After spawning, parental colonies were donated to the Palau Aquarium and displayed in the Reef Crest exhibit. Starting 20 hours after fertilisation, 50 % water changes were performed twice daily using 100 μm filters to siphon out water without disturbing the larvae, after which tanks were topped up with 0.2 µm UV-treated FSW. Gentle aeration through rigid air lines was introduced 36 hours after fertilisation when larvae became motile. Four days after fertilisation, mean larval survivorship was 77.0 ± 7.9 %, assessed using eight 50 ml aliquots. During settlement, twice-daily 50 % water changes were performed in each settlement tank, and aeration was added to maintain water movement. After one week, SUs were moved to the rearing tanks.

**Table S1** Statistical comparison of mean survival times (days) for juvenile coral colonies outplanted to the reef at six months old with five different levels of grazing deterrents monitored for 14 months (425 days).

| Treatment | Mean survival | 95% Confidence Interval | | |
| --- | --- | --- | --- | --- |
|  | time (days) | Lower bound | Upper bound |  |
| 4 long nails | 374 | 338.1 | 409.9 |  |
| 4 short nails | 354 | 319.5 | 388.5 |  |
| 2 long nails | 289 | 240.0 | 338.0 |  |
| 2 short nails | 324 | 277.7 | 370.3 |  |
| Control | 212 | 157.5 | 266.5 |  |

| Pairwise comparisons using Log-Rank test  Bonferroni *P* value adjustment | | | | |
| --- | --- | --- | --- | --- |
| Treatment | 4 long | 4 short | 2 long | 2 short |
| 4 short nails | 1.000 | - | - | - |
| 2 long nails | **0.042** | 0.760 | - | - |
| 2 short nails | 1.000 | 1.000 | 1.000 | - |
| Control | **<0.001** | **0.005** | 0.744 | **0.035** |

**Table S2** Percentage of replaced nails in each survey per treatment. Nails were only replaced for live corals, hence the number of replaced nails was divided over the number of nails present for all live corals to reach a percentage of nails. 160, 160, 80, 80 and 0 respectively.

| Survey | 1 | 2 | 3 | 4 | 5 | 6 | 7 | 8 | 9 | 10 |
| --- | --- | --- | --- | --- | --- | --- | --- | --- | --- | --- |
| Days after outplant | 0 | 7 | 35 | 62 | 98 | 140 | 160 | 227 | 341 | 425 |
| Days between surveys | NA | 7 | 28 | 27 | 36 | 42 | 20 | 67 | 114 | 84 |
| **Number of nails replaced** | | | | | | | | | | |
| 4 long nails | 0 | 2 | 24 | 13 | 11 | 19 | 7 | 70 | 31 | 15 |
| 4 short nails | 0 | 1 | 22 | 8 | 10 | 10 | 6 | 62 | 36 | 21 |
| 2 long nails | 0 | 0 | 7 | 4 | 0 | 5 | 4 | 21 | 3 | 4 |
| 2 short nails | 0 | 0 | 10 | 2 | 5 | 8 | 3 | 25 | 15 | 8 |
| Control | 0 | 0 | 0 | 0 | 0 | 0 | 0 | 0 | 0 | 0 |
| Total | 0 | 3 | 63 | 27 | 26 | 42 | 20 | 178 | 85 | 48 |
| **Percentage of nails replaced** | | | | | | | | | | |
| 4 long nails | 0% | 1.3% | 15.8% | 8.6% | 7.2% | 12.8% | 4.7% | 60.3% | 28.7% | 14.4% |
| 4 short nails | 0% | 0.6% | 14.5% | 5.5% | 6.6% | 6.6% | 3.9% | 55.4% | 42.9% | 26.3% |
| 2 long nails | 0% | 0% | 9.2% | 5.9% | 0% | 8.3% | 7.4% | 47.7% | 8.3% | 13.3% |
| 2 short nails | 0% | 0% | 13.9% | 2.8% | 7.4% | 12.5% | 4.8% | 54.3% | 35.7% | 20.0% |
| Control | 0% | 0% | 0% | 0% | 0% | 0% | 0% | 0% | 0% | 0% |
| Mean | 0% | 0.5% | 13.3% | 5.6% | 5.3% | 10.1% | 5.2% | 54.4% | 28.9% | 18.5% |

**Table S3** Model output for coral area data. The effect of treatment (five-levels fixed effect) on planar colony area after 14 months on the reef was tested using a Generalized Linear Mixed effects Model (GLMM; glmer, R Software) with a log link gamma distribution, accounting for variability between transects (four-level random factor). Significance codes represent p values as ‘***’ <0.001, ‘**’ <0.01, ‘*’ < 0.05, ‘.’ <0.1.

| **GLMM fit by maximum likelihood (Laplace Approximation)** | | | | | |
| --- | --- | --- | --- | --- | --- |
| Family: Gamma (log)  Formula: Area ~ Treatment + (1 \| Transect)  Number of observations: 89  Number of groups (Transect): 4 | | | | | |
|  | **Estimate** | **Std. Error** | **t value** | **p value** | **Signif. codes** |
| (Intercept) | 0.3058 | 0.3777 | 0.810 | 0.418080 |  |
| 4 long nails | 2.2783 | 0.4405 | 5.172 | 2.31e-07 | *** |
| 4 short nails | 2.1557 | 0.4548 | 4.740 | 2.14e-06 | *** |
| 2 long nails | 1.7533 | 0.4778 | 3.670 | 0.000243 | *** |
| 2 short nails | 1.5327 | 0.4548 | 3.370 | 0.000752 | *** |
| **Planned comparisons** |  |  |  |  |  |
| **Treatments** | **Estimate** | **Std. Error** | **z ratio** | **p value** | **Signif. codes** |
| Control - 4 long nails | -11.89 | 3.05 | -3.904 | 0.0009 | *** |
| Control - 4 short nails | -10.36 | 3.01 | -3.439 | 0.0053 | ** |
| Control - 2 long nails | -6.48 | 2.35 | -2.758 | 0.0460 | * |
| Control - 2 short nails | -4.93 | 1.67 | -2.946 | 0.0267 | * |
| 4 long nails - 4 short nails | 1.53 | 4.22 | 0.362 | 0.9963 |  |
| 4 long nails - 2 long nails | 5.41 | 3.78 | 1.432 | 0.6066 |  |
| 4 long nails - 2 short nails | 6.96 | 3.40 | 2.049 | 0.2429 |  |
| 4 short nails - 2 long nails | 3.88 | 3.75 | 1.035 | 0.8392 |  |
| 4 short nails - 2 short nails | 5.44 | 3.37 | 1.613 | 0.4892 |  |
| 2 long nails - 2 short nails | 1.55 | 2.79 | 0.556 | 0.9813 |  |

**Table S4** Model data for coral height data. The effect of treatment (five-levels fixed effect) on colony height after 14 months on the reef was tested using a GLMM (glmer, R Software) with a log link gamma distribution, accounting for variability between transects (four-level random factor). Significance codes represent p values as ‘***’ <0.001, ‘**’ <0.01, ‘*’ < 0.05, ‘.’ <0.1.

| **GLMM fit by maximum likelihood (Laplace Approximation)** | | | | | |
| --- | --- | --- | --- | --- | --- |
| Family: Gamma (log)  Formula: Height ~ Treatment + (1 \| Transect)  Number of observations: 91  Number of groups (Transect): 4 | | | | | |
|  | **Estimate** | **Std. Error** | **t value** | **p value** | **Signif. codes** |
| (Intercept) | -1.3471 | 0.3005 | -4.482 | 7.39e-06 | *** |
| 4 long nails | 2.1054 | 0.3536 | 5.953 | 2.63e-09 | *** |
| 4 short nails | 1.9293 | 0.3681 | 5.241 | 1.59e-07 | *** |
| 2 long nails | 1.6146 | 0.3880 | 4.161 | 3.17e-05 | *** |
| 2 short nails | 1.3959 | 0.3681 | 3.792 | 0.000149 | *** |
| **Planned comparisons** |  |  |  |  |  |
| **Treatments** | **Estimate** | **Std. Error** | **z ratio** | **p value** | **Signif. codes** |
| Control - 4 long nails | -1.875 | 0.405 | -4.623 | <.0001 | ******* |
| Control - 4 short nails | -1.530 | 0.388 | -3.940 | 0.0008 | ******* |
| Control - 2 long nails | -1.047 | 0.330 | -3.171 | 0.0132 | ***** |
| Control - 2 short nails | -0.790 | 0.236 | -3.341 | 0.0074 | ****** |
| 4 long nails - 4 short nails | 0.345 | 0.550 | 0.626 | 0.9709 |  |
| 4 long nails - 2 long nails | 0.828 | 0.511 | 1.620 | 0.4843 |  |
| 4 long nails - 2 short nails | 1.085 | 0.456 | 2.378 | 0.1214 |  |
| 4 short nails - 2 long nails | 0.483 | 0.498 | 0.972 | 0.8681 |  |
| 4 short nails - 2 short nails | 0.740 | 0.441 | 1.678 | 0.4477 |  |
| 2 long nails - 2 short nails | 0.257 | 0.391 | 0.657 | 0.9654 |  |

**Table S5** Model data for initial area loss. The effect of treatment (five-level fixed effect) on initial area loss, the percentage of planar colony area reduction during the first week after outplant, was tested using a gaussian GLMM (glmmTMB, R Software) and identity link accounting for differences between transects (four-level random factor). Significance codes represent p values as ‘***’ <0.001, ‘**’ <0.01, ‘*’ < 0.05, ‘.’ <0.1.

| **Generalized Linear Mixed effects Model** | | | | | |
| --- | --- | --- | --- | --- | --- |
| Family: gaussian (identity)  Formula: AreaPercentageReduction ~ Treatment + (1 \| Transect)  Dispersion: ~ Treatment  Number of observations: 194  Number of groups (Transect): 4 | | | | | |
| **Conditional model** | **Estimate** | **Std. Error** | **z value** | **p value** | **Signif. codes** |
| (Intercept) | 0.77813 | 0.06709 | 11.599 | < 2e-16 | *** |
| 4 long nails | -0.38240 | 0.04970 | -7.694 | 1.43e-14 | *** |
| 4 short nails | -0.39138 | 0.05691 | -6.877 | 6.11e-12 | *** |
| 2 long nails | -0.20460 | 0.05483 | -3.731 | 0.000191 | *** |
| 2 short nails | -0.21789 | 0.06166 | -3.534 | 0.000409 | *** |
| **Dispersion model** | **Estimate** | **Std. Error** | **z value** | **p value** | **Signif. codes** |
| (Intercept) | -1.56770 | 0.12110 | -12.946 | <2e-16 | *** |
| 4 long nails | 0.08728 | 0.17111 | 0.510 | 0.6100 |  |
| 4 short nails | 0.32036 | 0.16823 | 1.904 | 0.0569 | . |
| 2 long nails | 0.26067 | 0.16385 | 1.591 | 0.1116 |  |
| 2 short nails | 0.40161 | 0.16724 | 2.401 | 0.0163 | * |

| **Planned comparisons** |  |  |  |  |  |  |
| --- | --- | --- | --- | --- | --- | --- |
| **Treatments** | **Estimate** | **Std. Error** | **df** | **t ratio** | **p value** | **Signif. codes** |
| Control - 4 long nails | 0.38240 | 0.0497 | 183 | 7.694 | <.0001 | ******* |
| Control - 4 short nails | 0.39138 | 0.0569 | 183 | 6.877 | <.0001 | ******* |
| Control - 2 long nails | 0.20460 | 0.0548 | 183 | 3.731 | 0.0023 | ****** |
| Control - 2 short nails | 0.21789 | 0.0617 | 183 | 3.534 | 0.0047 | ****** |
| 4 long nails - 4 short nails | 0.00899 | 0.0579 | 183 | 0.155 | 0.9999 |  |
| 4 long nails - 2 long nails | -0.17780 | 0.0559 | 183 | -3.180 | 0.0147 | ***** |
| 4 long nails - 2 short nails | -0.16450 | 0.0626 | 183 | -2.627 | 0.0697 | **.** |
| 4 short nails - 2 long nails | -0.18678 | 0.0624 | 183 | -2.993 | 0.0258 | ***** |
| 4 short nails - 2 short nails | -0.17349 | 0.0685 | 183 | -2.533 | 0.0878 | **.** |
| 2 long nails - 2 short nails | 0.01329 | 0.0668 | 183 | 0.199 | 0.9996 |  |

**Table S6** Model data for the predicted relationship between the initial area loss, percentage of planar colony area reduction during the first week after outplant on survival after 14 months, was tested using a GLMM (glmmTMB, R Software) with a binominal error distribution with treatments and transects as random effects. Significance codes represent p values as ‘***’ <0.001, ‘**’ <0.01, ‘*’ < 0.05, ‘.’ <0.1.

| **Generalized Linear Mixed effects Model** | | | | | |
| --- | --- | --- | --- | --- | --- |
| Family: binomial (logit)  Formula: Alive14m ~ AreaPercentageReduction + (1 \| Treatment) + (1 \| Transect)  Number of observations: 164  Number of groups: Treatment, 5; Transect, 4 | | | | | |
|  | **Estimate** | **Std. Error** | **z value** | **p value** | **Signif. codes** |
| (Intercept) | 1.6472 | 0.4216 | 3.907 | 9.36e-05 | *** |
| AreaPercentageReduction | -2.7696 | 0.6369 | -4.349 | 1.37e-05 | *** |

**Table S7** Model data for bite rate analysis. The effect of each grazing deterrent treatment on fish bite rates, of all fish species grouped, was tested using a GLMM (glmmTMB, R Software) with a negative binomial error distribution with plot as random effect (three-level random factor), as data distribution was overdispersed when first run with a Poisson error distribution. Significance codes represent p values as ‘***’ <0.001, ‘**’ <0.01, ‘*’ < 0.05, ‘.’ <0.1.

| **Generalized Linear Mixed effects Model** | | | | | |
| --- | --- | --- | --- | --- | --- |
| Family: nbinom2 (log)  Formula: BitesH.c ~ GrazingDeterrent + (1 \| Plot)  Number of observations: 105  Number of groups (Plot): 3  Dispersion parameter: 0.247  Results are given on the log (not the response) scale.  P value adjustment: tukey method for comparing a family of 5 estimates | | | | | |
|  | **Estimate** | **Std. Error** | **z value** | **p value** | **Signif. codes** |
| (Intercept) | 0.8932 | 0.6431 | 1.389 | 0.16488 |  |
| 4 long nails | -1.0178 | 0.7163 | -1.421 | 0.15536 |  |
| 4 short nails | -2.3405 | 0.7844 | -2.984 | 0.00285 | ** |
| 2 long nails | 0.1054 | 0.6521 | 0.162 | 0.87156 |  |
| 2 short nails | -0.5200 | 0.6803 | -0.764 | 0.44470 |  |
| **Planned comparisons** |  |  |  |  |  |
| **Treatments** | **Estimate** | **Std. Error** | **z ratio** | **p value** | **Signif. codes** |
| Control - 4 long nails | 1.018 | 0.716 | 1.421 | 0.6141 |  |
| Control - 4 short nails | 2.341 | 0.784 | 2.984 | 0.0238 | ***** |
| Control - 2 long nails | -0.105 | 0.652 | -0.162 | 0.9998 |  |
| Control - 2 short nails | 0.520 | 0.680 | 0.764 | 0.9408 |  |
| 4 long nails - 4 short nails | 1.323 | 0.829 | 1.596 | 0.5001 |  |
| 4 long nails - 2 long nails | -1.123 | 0.728 | -1.542 | 0.5348 |  |
| 4 long nails - 2 short nails | -0.498 | 0.720 | -0.691 | 0.9585 |  |
| 4 short nails - 2 long nails | -2.446 | 0.786 | -3.112 | 0.0160 | ***** |
| 4 short nails - 2 short nails | -1.821 | 0.808 | -2.255 | 0.1599 |  |
| 2 long nails - 2 short nails | 0.625 | 0.687 | 0.910 | 0.8932 |  |

**Fig. S1** Size frequency distribution of fishes observed grazing on the additional outplants for grazing assays. Every count was an individual grazing event, defined as a sequence of consecutive bites by a single fish without the fish leaving the frame.

**Table S8** Grazing events recorded in the video assays. Time is the time of camera deployment on the reef, size is the size of the observed fish to the nearest 5 cm, and bites are the number of bites taken by a single fish. The maturation phases are initial phase (IP) and juvenile phase (JP).

| # | Date | Time | Plot | Treatment | Species | Group | Size | Bites | Phase |
| --- | --- | --- | --- | --- | --- | --- | --- | --- | --- |
| 1 | 16/4/22 | 10:47 | 2 | 2 long | *Plectroglyphidodon dickii* | Damselfish | 5 | 1 |  |
| 2 | 16/4/22 | 10:47 | 2 | 2 long | *Chlorurus spilurus* | Parrotfish | 15 | 3 | IP/JP |
| 3 | 16/4/22 | 10:47 | 2 | 2 long | *Chlorurus spilurus* | Parrotfish | 15 | 2 | IP/JP |
| 4 | 16/4/22 | 10:47 | 2 | 2 long | *Chlorurus spilurus* | Parrotfish | 15 | 5 | IP/JP |
| 5 | 16/4/22 | 10:47 | 2 | 2 long | *Ctenochaetus striatus* | Surgeonfish | 15 | 6 |  |
| 6 | 16/4/22 | 10:47 | 2 | 2 short | *Chlorurus spilurus* | Parrotfish | 15 | 2 | IP/JP |
| 7 | 16/4/22 | 10:47 | 2 | 2 short | *Chlorurus spilurus* | Parrotfish | 15 | 5 | IP/JP |
| 8 | 16/4/22 | 10:47 | 2 | 2 short | *Chlorurus spilurus* | Parrotfish | 15 | 1 | IP/JP |
| 9 | 16/4/22 | 10:47 | 2 | 4 long | *Chlorurus spilurus* | Parrotfish | 15 | 1 | IP/JP |
| 10 | 16/4/22 | 10:47 | 2 | 4 long | *Chlorurus spilurus* | Parrotfish | 15 | 2 | IP/JP |
| 11 | 16/4/22 | 10:47 | 2 | 4 short | *Chlorurus spilurus* | Parrotfish | 15 | 5 | IP/JP |
| 12 | 16/4/22 | 10:47 | 2 | Control | *Chlorurus spilurus* | Parrotfish | 20 | 1 | IP/JP |
| 13 | 16/4/22 | 10:47 | 2 | Control | *Chlorurus spilurus* | Parrotfish | 15 | 8 | IP/JP |
| 14 | 16/4/22 | 10:47 | 2 | Control | *Chlorurus spilurus* | Parrotfish | 15 | 6 | IP/JP |
| 15 | 16/4/22 | 10:47 | 2 | Control | *Chlorurus spilurus* | Parrotfish | 15 | 3 | IP/JP |
| 16 | 16/4/22 | 10:47 | 2 | Control | *Chlorurus spilurus* | Parrotfish | 15 | 1 | IP/JP |
| 17 | 16/4/22 | 10:47 | 2 | Control | *Chlorurus spilurus* | Parrotfish | 15 | 2 | IP/JP |
| 18 | 16/4/22 | 10:47 | 2 | Control | *Chlorurus spilurus* | Parrotfish | 15 | 1 | IP/JP |
| 19 | 17/4/22 | 09:44 | 1 | Control | *Labrichthys unilineatus* | Wrasse | 10 | 2 |  |
| 20 | 17/4/22 | 09:44 | 1 | Control | *Labrichthys unilineatus* | Wrasse | 10 | 12 |  |
| 21 | 17/4/22 | 09:44 | 1 | Control | *Halichoeres melanurus* | Wrasse | 5 | 1 | IP |
| 22 | 17/4/22 | 09:44 | 1 | Control | *Halichoeres melanurus* | Wrasse | 5 | 2 |  |
| 23 | 17/4/22 | 09:28 | 2 | 2 long | *Chlorurus spilurus* | Parrotfish | 10 | 2 |  |
| 24 | 17/4/22 | 09:28 | 2 | 2 long | *Chlorurus spilurus* | Parrotfish | 10 | 1 |  |
| 25 | 17/4/22 | 09:35 | 3 | 4 long | *Halichoeres hortulanus* | Wrasse | 10 | 1 |  |
| 26 | 17/4/22 | 12:16 | 1 | 2 long | *Labrichthys unilineatus* | Wrasse | 10 | 5 | IP |
| 27 | 17/4/22 | 12:16 | 1 | 2 long | *Labrichthys unilineatus* | Wrasse | 10 | 8 | IP |
| 28 | 17/4/22 | 12:16 | 1 | 2 long | *Labrichthys unilineatus* | Wrasse | 10 | 9 | IP |
| 29 | 17/4/22 | 12:16 | 1 | 2 long | *Labrichthys unilineatus* | Wrasse | 10 | 4 | IP |
| 30 | 17/4/22 | 12:16 | 1 | 2 long | *Labrichthys unilineatus* | Wrasse | 10 | 13 | IP |
| 31 | 17/4/22 | 12:16 | 1 | 2 long | *Labrichthys unilineatus* | Wrasse | 10 | 17 | IP |
| 32 | 17/4/22 | 12:16 | 1 | 2 long | *Labrichthys unilineatus* | Wrasse | 10 | 7 | IP |
| 33 | 17/4/22 | 12:16 | 1 | 2 long | *Labrichthys unilineatus* | Wrasse | 10 | 5 | IP |
| 34 | 17/4/22 | 12:16 | 1 | 2 long | *Labrichthys unilineatus* | Wrasse | 10 | 3 | IP |
| 35 | 17/4/22 | 12:16 | 1 | 2 long | *Labrichthys unilineatus* | Wrasse | 10 | 7 | IP |
| 36 | 17/4/22 | 12:16 | 1 | 2 short | *Labrichthys unilineatus* | Wrasse | 10 | 13 | IP |
| 37 | 17/4/22 | 12:16 | 1 | 2 short | *Labrichthys unilineatus* | Wrasse | 10 | 14 | IP |
| 38 | 17/4/22 | 12:16 | 1 | Control | *Labrichthys unilineatus* | Wrasse | 10 | 5 | IP |
| 39 | 17/4/22 | 12:16 | 1 | Control | *Labrichthys unilineatus* | Wrasse | 10 | 7 | IP |
| 40 | 17/4/22 | 12:16 | 1 | Control | *Labrichthys unilineatus* | Wrasse | 10 | 6 | IP |
| 41 | 17/4/22 | 12:16 | 1 | Control | *Labrichthys unilineatus* | Wrasse | 10 | 2 | IP |
| 42 | 17/4/22 | 12:16 | 1 | Control | *Labrichthys unilineatus* | Wrasse | 10 | 7 | IP |
| 43 | 17/4/22 | 12:19 | 2 | 2 long | *Apogon exostigma* | Cardinal fish | 5 | 1 |  |
| 44 | 17/4/22 | 12:19 | 2 | 2 short | *Chaetodon citrinellus* | Butterflyfish | 10 | 1 |  |
| 45 | 17/4/22 | 15:10 | 1 | Control | *Labrichthys unilineatus* | Wrasse | 10 | 2 |  |
| 46 | 17/4/22 | 15:10 | 1 | Control | *Labrichthys unilineatus* | Wrasse | 10 | 3 | IP |
| 47 | 17/4/22 | 15:06 | 3 | 2 short | *Chaetodon citrinellus* | Butterflyfish | 5 | 2 |  |
| 48 | 17/4/22 | 15:06 | 3 | Control | *Balistapus undulatus* | Triggerfish | 15 | 1 |  |
| 49 | 18/4/22 | 09:13 | 1 | 2 long | *Ctenochaetus striatus* | Surgeonfish | 10 | 1 |  |
| 50 | 18/4/22 | 09:13 | 1 | 2 long | *Labrichthys unilineatus* | Wrasse | 10 | 3 | IP |
| 51 | 18/4/22 | 09:13 | 1 | 4 short | *Chaetodon baronessa* | Butterflyfish | 10 | 2 |  |
| 52 | 18/4/22 | 09:09 | 2 | 4 long | *Chlorurus spilurus* | Parrotfish | 15 | 3 | JP |
| 53 | 18/4/22 | 09:04 | 3 | 2 long | *Ctenochaetus striatus* | Surgeonfish | 15 | 1 |  |
| 54 | 18/4/22 | 09:04 | 3 | 2 short | *Chaetodon citrinellus* | Butterflyfish | 10 | 2 |  |
| 55 | 18/4/22 | 09:04 | 3 | Control | *Thalassoma quinquevittatum* | Wrasse | 5 | 2 |  |
| 56 | 18/4/22 | 11:40 | 1 | 2 long | *Labrichthys unilineatus* | Wrasse | 10 | 2 |  |
| 57 | 18/4/22 | 11:40 | 1 | 2 long | *Labrichthys unilineatus* | Wrasse | 10 | 6 |  |
| 58 | 18/4/22 | 11:40 | 1 | 4 long | *Thalassoma hardwicke* | Wrasse | 5 | 1 |  |
| 59 | 18/4/22 | 11:40 | 1 | 4 long | *Thalassoma hardwicke* | Wrasse | 5 | 1 |  |
| 60 | 18/4/22 | 11:40 | 1 | Control | *Labrichthys unilineatus* | Wrasse | 10 | 1 |  |
| 61 | 18/4/22 | 11:42 | 2 | 2 long | *Halichoeres melanurus* | Wrasse | 5 | 1 |  |
| 62 | 18/4/22 | 11:42 | 2 | 2 short | *Chaetodon citrinellus* | Butterflyfish | 10 | 1 |  |
| 63 | 18/4/22 | 11:42 | 2 | 4 long | *Halichoeres melanurus* | Wrasse | 5 | 1 |  |
| 64 | 18/4/22 | 11:42 | 2 | 4 long | *Chlorurus spilurus* | Parrotfish | 10 | 4 | IP/JP |
| 65 | 18/4/22 | 11:42 | 2 | 4 long | *Halichoeres melanurus* | Wrasse | 5 | 1 |  |
| 66 | 18/4/22 | 11:42 | 2 | 4 long | *Chlorurus spilurus* | Parrotfish | 15 | 1 | IP/JP |
| 67 | 18/4/22 | 11:42 | 2 | 4 long | *Halichoeres marginatus* | Wrasse | 10 | 1 |  |
| 68 | 18/4/22 | 11:44 | 3 | 2 short | *Ctenochaetus striatus* | Surgeonfish | 10 | 3 |  |
| 69 | 18/4/22 | 11:44 | 3 | 4 long | *Scarus psittacus* | Parrotfish | 15 | 2 | IP |
| 70 | 18/4/22 | 11:44 | 3 | 4 long | *Scarus psittacus* | Parrotfish | 15 | 4 | IP |
| 71 | 18/4/22 | 11:44 | 3 | Control | *Scarus psittacus* | Parrotfish | 15 | 1 | IP |
| 72 | 18/4/22 | 14:30 | 1 | 2 long | *Chaetodon baronessa* | Butterflyfish | 10 | 3 |  |
| 73 | 18/4/22 | 14:30 | 1 | 2 short | *Chaetodon baronessa* | Butterflyfish | 10 | 2 |  |
| 74 | 18/4/22 | 14:30 | 1 | 4 long | *Chaetodon baronessa* | Butterflyfish | 10 | 6 |  |
| 75 | 18/4/22 | 14:30 | 1 | Control | *Chaetodon baronessa* | Butterflyfish | 10 | 11 |  |
| 76 | 18/4/22 | 14:32 | 2 | 2 long | *Chlorurus spilurus* | Parrotfish | 10 | 3 |  |
| 77 | 18/4/22 | 14:32 | 2 | Control | *Halichoeres melanurus* | Wrasse | 5 | 2 |  |
